# Semaphorin-3A as An Immune Modulator Is Suppressed by MicroRNA-145-5p

**DOI:** 10.22074/cellj.2018.4842

**Published:** 2018-01-01

**Authors:** Mahsa Rezaeepoor, Mazdak Ganjalikhani-hakemi, Shima Shapoori, Nahid Eskandari, Mohammadreza Sharifi, Masoud Etemadifar, Mansuorian Marjan

**Affiliations:** 1 Department of Immunology, Faculty of Medicine, Isfahan University of Medical Sciences, Isfahan, Iran; 2 Department of Genetics, Faculty of Medicine, Isfahan University of Medical Sciences, Isfahan, Iran; 3 Multiple Sclerosis and Neuroimmunology Research Center, Isfahan University of Medical Sciences, Isfahan, Iran; 4 Department of Biostatistics and Epidemiology, Faculty of Medicine, Isfahan University of Medical Science, Isfahan, Iran

**Keywords:** microRNAs, miR-145-5p, Semaphorin-3A

## Abstract

**Objective:**

Semaphorin-3A (SEMA3A) and its receptors are found on some immune cells and act as suppressors of immune cells over-activation. Considering the role of *SEMA3A* and its down-regulation in some autoimmune diseases, as well as our bioinformatics predictions, we assumed that miR-145-5p might affect *SEMA3A* expression. So, we aimed to determine the effect of miR-145-5p on *SEMA3A* gene expression level.

**Materials and Methods:**

In this experimental study, we evaluated the effect of miR-145-5p transfection on *SEMA3A* expression in peripheral blood mononuclear cells (PBMCs) using ELISA and quantitative real-time polymerase chain reaction (PCR) methods.

**Results:**

Our results showed that miR-145-5p is able to decrease *SEMA3A* expression at both protein and mRNA levels. These data confirmed our previous bioinformatic prediction about the inhibitory effect of miR-145-5p on *SEMA3A* expression.

**Conclusion:**

These results enlightened us about an unknown aspect of *SEMA3A* role in some autoimmune disorders like multiple sclerosis (MS) and rheumatoid arthritis (RA) and also proposed *SEMA3A* as a potential therapeutic approach.

## Introduction

Semaphorins are members of a large and diverse protein family and contain a common conserved cysteinerich domain located in N-terminal region. This domain includes approximately 500 amino acids in length and is termed "sema" domain (1). Semaphorins could be seen as secreted transmembrane or glycosyl phosphatidyl inositol (GPI)-linked proteins and up to now, 20 types of them have been observed in humans (2). 

Semaphorins were first found in 1990s as axon guidance molecules which could act as bifunctional signaling molecules to supply chemorepellent or chemoattractant cues in the nervous system (1). Semaphorin 3A (SEMA3A) which belongs to semaphorins family, was the first semaphorin discovered in vertebrate in 1993 (3). Sema3-A is a secreted protein that has prominent roles in regulation of the immune system and has been found to be correlated with some autoimmune diseases.

*SEMA3A* is highly expressed in activated CD4+ T cells and while it is expressed at lower levels in CD8+ T cells. SEMA3A secretion is delayed, so it seems that SEMA3A has a determining role in terminating the immune responses (4). Also, it has been found that mature dendritic cell (DC) and differentiating macrophages produce high amounts of SEMA3A but immature DCs secrete lower levels of SEMA3A (4, 5). In addition, B regulatory cells (CD25 and CD86) and T regulatory cells express high levels of *SEMA3A*. It was suggested that SEMA3A could be considered as a B regulatory cells marker (6, 7).

SEMA3A is able to convey its signal through plexin A1 or plexin A4 by direct binding to neuropilin-1 with high affinity. Previously, high expression of *NPR1* in T regulatory cells was also reported (8, 9). 

The main function of SEMA3A in the immune system is modulation of immune responses. SEMA3A can suppress B and T cell proliferation and alleviate generation of pro-inflammatory cytokines such as tumour necrosis factor-alpha (TNF-a) and interferon- gamma (IFN-.) by T cells (4). Also, it enhances the regulatory properties of B cells and induces apoptosis in monocyte-derived macrophage colosny-stimulating factor (M-CSF)-differentiated macrophages (6, 10). 

Furthermore, microRNAs (MiRNAs) were also discovered in 1993 (11). Amajor part of the human genome is transcribed, but only around 2% of these transcripts are translated into proteins. Many of the remaining transcripts which are not translated into protein, are RNA molecules and have biological functions. These RNA molecules have been classified based on their sizes and non-coding RNAs (ncRNAs) which are less than 200 nucleotides in length are called short non-coding RNAs (sncRNAs) (12). 

MiRNAs which belong to sncRNAs have 19-22 nucleotides in length and are posttranscriptional regulatory RNA molecules that participate in the regulation of gene expression through base pairing with 3' untranslated regions (3'UTR) of mRNAs. This binding leads to mRNA instability (13). MiRNAs could participate in the regulation of apoptosis, hematopoiesis, immune regulation and other biological processes (14, 15). Recently, it has been revealed that miRNAs participate in a broad spectrum of human diseases such as neurological disorders.

*MicroRNA-145* gene which is located on chromosome 5, is a member of the miR-143/145 cluster (16). It seems that primary miRNA (pri-microRNA) structure of miR-145-5p is co-transcribed with miR-143. Reduction of miR-145 has been detected in multiple tumors including breast, pancreas, prostate and colon. Actually, miR-145 has been shown as a tumor- suppressor gene because of its pro-apoptotic and anti- proliferative properties (17). MiR-145 negatively regulates oncogenes participating in cell proliferation and survival. Noteworthy, it has been demonstrated that p53 can induce miR-145 transcription in response to anticancer drugs and serum starvation (18). Also, miR-145 is able to suppress metastasis of breast cancer cells by targeting mucin-1 (19). In addition, as reported by Fayyad-Kazan et al. (20), miR-145 is down- regulated in T regulatory cells and it can negatively regulate *CTLA-4M* expression in CD4+ regulatory T cells in human adults.

Moreover, alteration of miR-145-5p was investigated in multiple sclerosis (MS) and it has been demonstrated that miR-145-5p is overexpressed in peripheral blood mononuclear cells (PBMCs) of MS patients. In MS patients, miR-145-5p is overexpressed (3-folds) as compared to controls (21, 22).

As previously described, SEMA3A is produced by PBMCs such as activated lymphocytes and plays anti-inflammatory roles in the immune system. The role and alteration in the level of SEMA3A have been investigated in some autoimmune diseases, including systemic lupus erythematosus (SLE), rheumatoid arthritis (RA), psoriasis and systemic sclerosis (SSc). In some of these studies, down-regulation of SEMA3A have been reported (7, 23-25). 

Based on our bioinformatic predictions (using Targetscan and miRwalk softwares), we assumed that miR-145-5p could have a possible powerful interaction with *SEMA3A*. Therefore, decreased expression of SEMA3A might be correlated with enhancement of miR-145-5p in some autoimmune diseases. 

In this study, we aimed to investigate the relationship between the inhibitory effect of miR-145-5p and *SEMA3A* gene expression. This could be considered as a possible therapeutic/diagnostic approach against autoimmune diseases using miR-145-5p. 

## Materials and Methods

Firstly, Mirwalk 2.0 (http://zmf.umm.uni-heidelberg. de/apps/zmf/mirwalk2/) was used to make bioinformatic prediction of *miRNA-SEMA3A* interaction. In the “predicted targets module”, we selected “Gene-miRNA target” and then, put the *SEMA3A* RefSeq ID (NM_ 006080.2) in the relevant box. Input parameters were adjusted for finding 3´-UTR and all databases were chosen. After that, Target Scan 7.0 (http://www.targetscan. org/) was used to confirm bioinformatic prediction made by Mirwalk. We put the gene symbol of SEMA3A in the relevant box and then, submitted the query.

### Cell isolation and culture

In this experimental study, PBMCs were isolated fromhealthy donors using Ficoll-Isopaque (Lymphodex, Germany). The study was approved by the EthicsCommittee of Isfahan University of Medical Sciencesof Iran, Isfahan, Iran. Then, cells were cultured in RPMI 1640 [(Biosera, France), containing 10% fetal bovine serum (FBS, Biosera, France)] and 1% penicillin- streptomycin (Biosera, France) in 6-well U-bottomplates (1×10^6^ cells/well/2 ml) in the presence of 7.5 µL/ml Phytohemagglutinin (PHA, Sigma-Aldrich, Germany). Cells were incubated for 96-144 hours at37°C in a humidified chamber with 5% CO_2_ (Memert, Germany). 

### Transfection 

**Fig.1:**
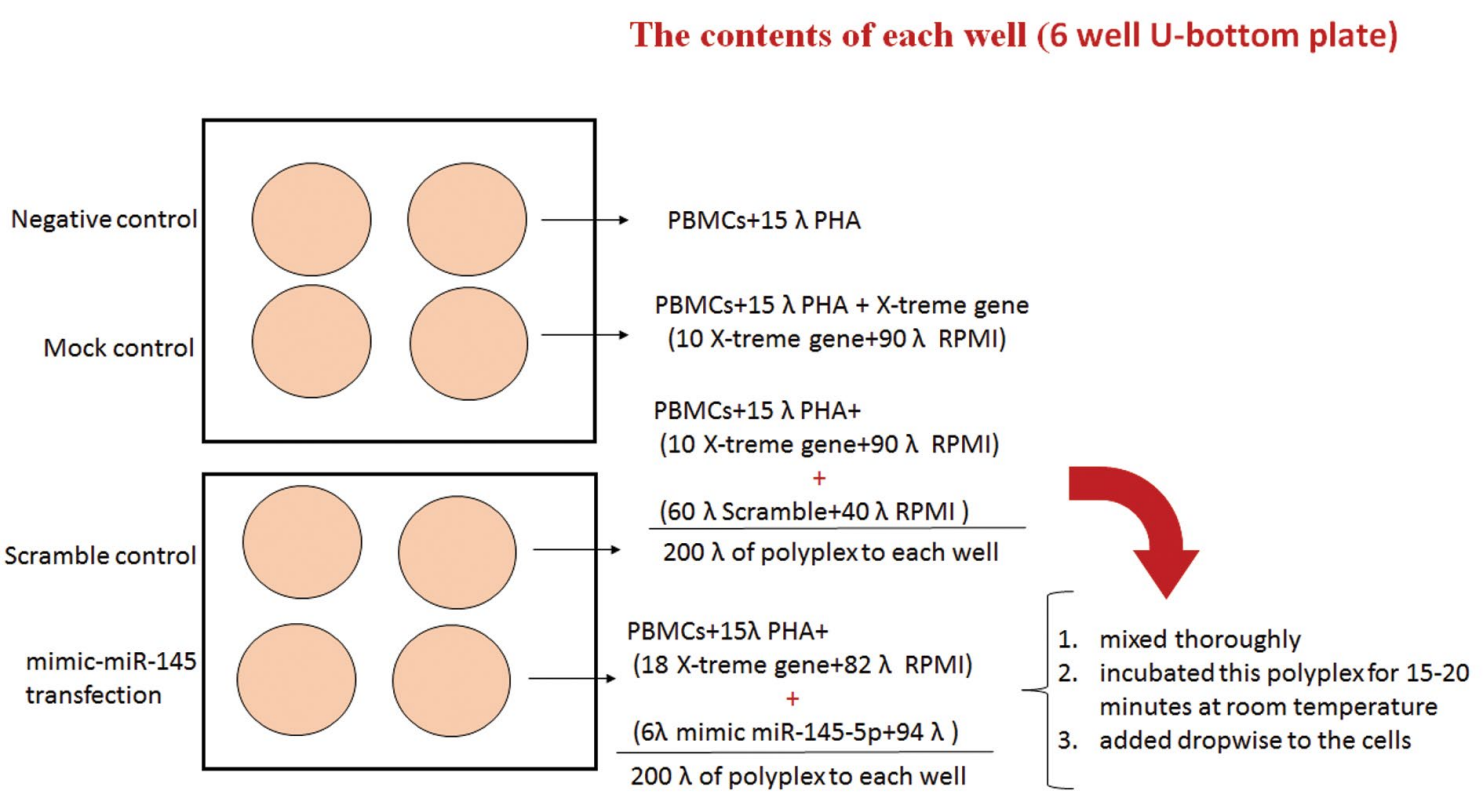
Control was peripheral blood mononuclear cells (PBMCs), which stimulated with Phytohemagglutinin (PHA); mock control was PBMCs stimulated with PHA and treated with X-treme gene reagent; Scramble (Scr) which is a transfection of negative control SiRNA, was comprised of PBMCs treated with PHA, X-treme gene reagent and Lable IT RNAi Delivery Control; miR-145-5p, PBMCs stimulated with PHA and treated with X-treme gene reagent and mimic-miR-145-5p.

After 72 hours, cultured cells were transfected (50 nM final concentration) with miR-145-5p mimic (Qiagen, Germany) using X-treme gene (Roche, Germany). The culture medium was refreshed and cell culture was continued for 24 hours. Treatment with X-treme gene alone was applied as mock control. PBMCs in some wells were transfected with Label IT® RNAi Delivery Control (Mirus, USA) both as an indicator of transfection efficiency and as a scrambled siRNA (Fig .1). Transfection efficiency was analyzed by FACS Calibour flow cytometer (Becton Dickinson) and CellQuest^TM^ Pro software.

### Quantitative real-time polymerase chain reaction analysis of SEMA3A mRNA level

Total RNA was isolated from PBMCs using RNXTMPLUS (CinnaGen, Iran) and it was reverse transcribed using a first-strand cDNA synthesis kit (Thermo scientific, USA) according to the manufacturer’s instructions. Sense and antisense primers were:

5´-TGTTGGGACCGTTCTTAAAGTAGT-3´ and 5´-TAGTTGTTGCTGCTTAGTGGAAAG-3´for SEMA3A and 5´-TGAAGATCAAGATCATTGCTCCTC-3´ and 5´-CAACTAAGTCATAGTCCGCCTAGA-3´ for *ß-actin* as the housekeeping gene. Real-time polymerase chain reaction (PCR) analysis was done using miScript SYBR green PCR kit (Thermo fisher, USA) and a StepOnePlus device (Applied Biosystems, USA). For quantitative RT-PCR (qRT-PCR) analysis, 1 cycle at 95°C for 10 minutes and then, 40 cycles at 95°C for 15 seconds and at 61°C for 1 minute, were done. The relative miRNA expression was calculated using the 2^-ΔΔCt^ method.

### SEMA3A protein assay

Cells were centrifuged and an aliquot of the supernatant was collected for SEMA3A level analysis by ELISA. The level of secreted SEMA3A was evaluated by a commercial ELISA kit (Elabscience, China) according to the manufacturer’s instructions.

### Methylthiazole tetrazolium assay 

The MTT assay has been used as a rapid and sensitive method for assessment of chemicals’ cytotoxicity. Here, 90 µl cell-containing medium was added to each well of a 96well plate and then, 10 µl MMT solution was added to each well. Cells were incubated for 1 hour at 37°C with 5% CO_2_.Then, the medium was removed and the plate was frozen for 1 hour at -80°C. Then, 100µl dimethyl sulfoxide (DMSO, Parstous, Iran) was added to each well and incubated for 30 minutes at 37°C while shaking. Finally, the optimal density of each well was measured at 590 nm.

### Statistical analysis 

For statistical analyses, SPSS 20.0 software was used. One Way ANOVA was utilized for making comparisons between treated groups. All experiments were performed in triplicate. Data are expressed as mean ± SD, and P<0.05 were considered statistically significant.

## Results

### The miR-145-5p was predicted as an SEMA3A silencer miRNA 

**Fig.2:**
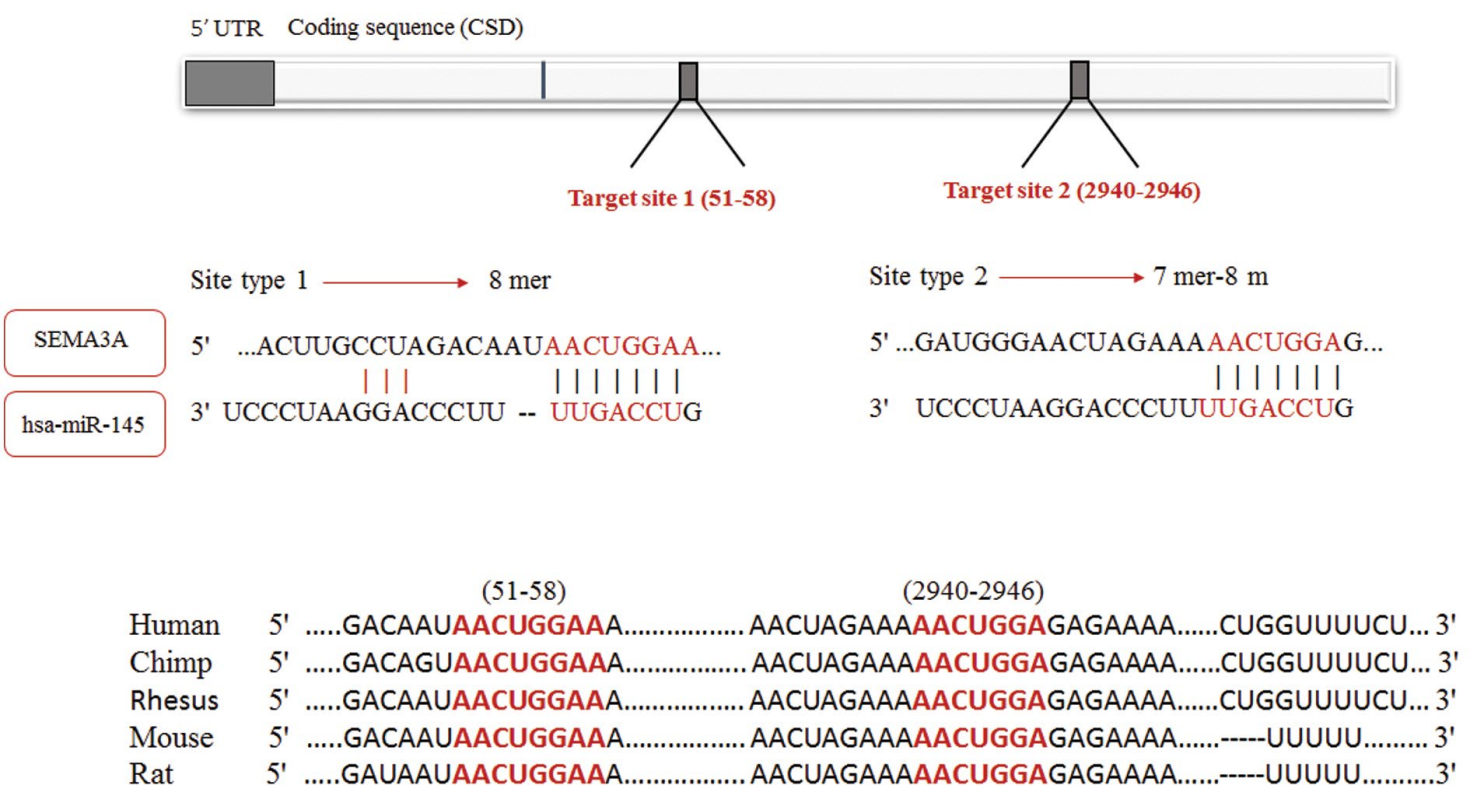
Predicted hsa-miR-145-5p complementary sequence in *SEMA3A* mRNA 3´-UTR and its conservation status. A. Indicates the positions of predicted complementary sequence located in *SEMA3A* mRNA 3´-UTR and also pairing status of hsa-miR-145-5p and predicted target sequence and B. Indicates conservation status of predicted complementary sequence in human and other mammals.

According to the Mirwalk output, miR-145-5p was predicted to suppress *SEMA3A* expression by 7/12 of selected algorithms (miRWalk, miRanda, miRDB, PICTAR2, PITA, RNA hybrid and Targetscan). “Validated targets module” showed nothing for miR145- 5p-SEMA3A interaction which represented that no experimental study had been done to validate this bioinformatic prediction. In the Targetscan output, a context score percentile of 99% was predicted for miR145- 5p for silencing *SEMA3A* expression with an 8-mer seed region (Fig .2).

### Efficiency of transfection 

**Fig.3:**
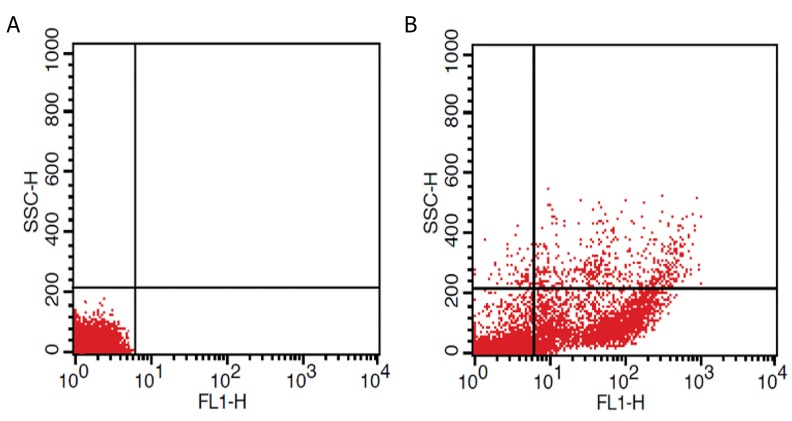
Flow cytometry analysis of peripheral blood mononuclear cells (PBMCs) after transfection with FITC labeled siRNA. As indicated in dot plot diagram, incompared with the A. Negative control and B. Transfection efficiency was 81%.

Incubation with 50 nM final concentration of Lable IT siRNA Delivery Control-FITC for 24 hour, revealed that 81% of the cells were successfully transfected (Fig .3).

### Decreased expression of *SEMA3A* in PBMCs in the presence of miR-145-5p

**Fig.4:**
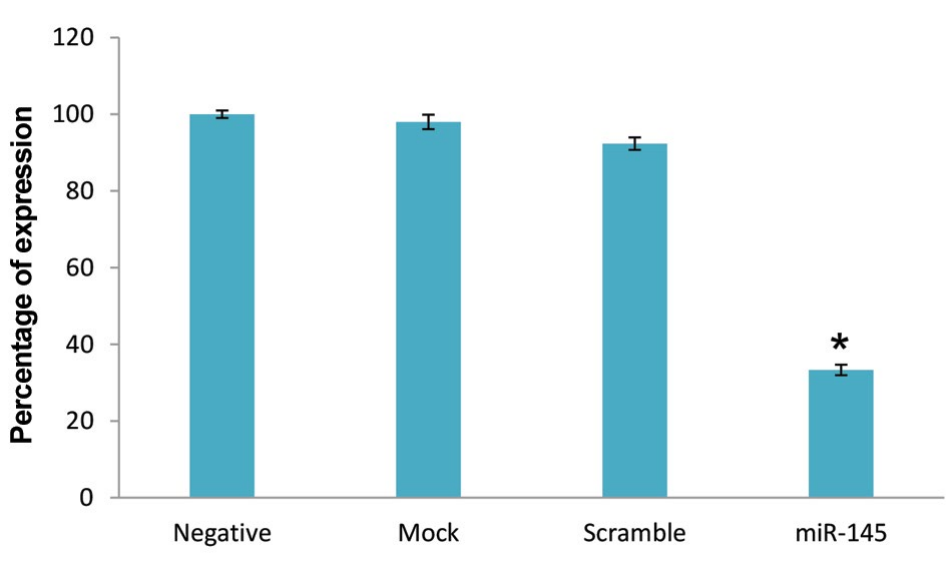
Quantitative real time polymerase chain reaction (PCR) analysis was performed to determine *SEMA3A* expression level in peripheral bloodmononuclear cells (PBMCs) before and after transfection of miR-145-5p mimic. No statistically significant difference was demonstrated between control groups. Transfection of miR-145-5p mimic result in decrease of *SEMA3A* expression. Enhancement in miR-145-5p was associated significantly with down-regulation of SEMA3A (*; P<0.05).

To investigate *SEMA3A* mRNA expression, we performed quantitative real-time PCR analysis. In the transfected cells with miR-145-5p mimic, *SEMA3A* transcript level was highly down-regulated as compared to controls. This decrease in *SEMA3A* transcript level was statistically significant (P=0.0001) in comparison to negative control. Mock and scrambled groups showed no meaningful reduction in *SEMA3A* expression level compared to the negative control (Fig .4).

### SEMA3A secretion was down-regulated after miR145- 5p transfection 

**Fig.5:**
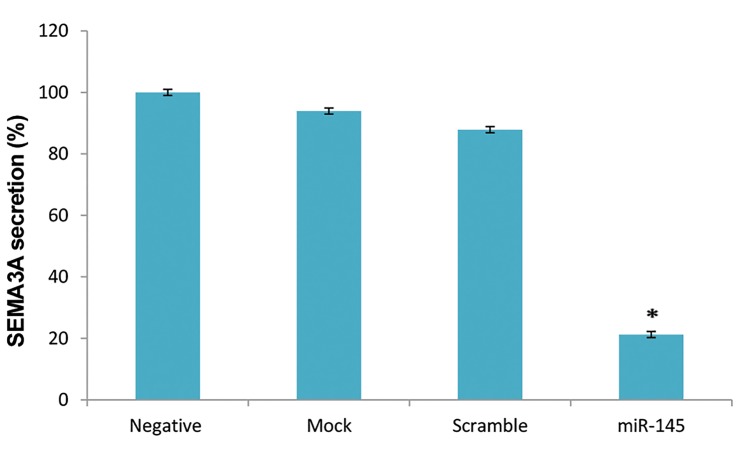
Investigation of SEMA3A secretion from peripheral blood mononuclear cells (PBMCs) was performed by ELISA assay. No statistically significant difference was observed between un-transfected cells and mock/scrambled groups. Transfection of miR-145-5p mimic results in down regulation of SEMA3A and there was significantly difference between transfected cell and other control groups (*; P<0.05). Control was PBMCs, which stimulated with Phytohemagglutinin (PHA); mock control was PBMCs stimulated with PHA and treated with X-treme gene reagent; Scramble (Scr) which is a transfection of negative control SiRNA, was comprised of PBMCs treated with PHA, X-treme gene reagent and Lable IT RNAi Delivery Control; miR-145-5p, PBMCs stimulated with PHA and treated with X-treme gene reagent and mimic-miR-145-5p.

Based on the ELISA results, the SEMA3A secretion by cells transfected with miR-145-5p mimic was lower than other groups (Fig.5). There was no significant difference among negative control, mock and scrambled groups. SEMA3A level was considerably lower in miR-145-5ptransfected cells (0.16 ± 0.01 ng/ml) compared to control cells (0.79 ± 0.01 ng/ml) and this decrease was significant (P=0.015). 

### Cell viability assay 

**Fig.6:**
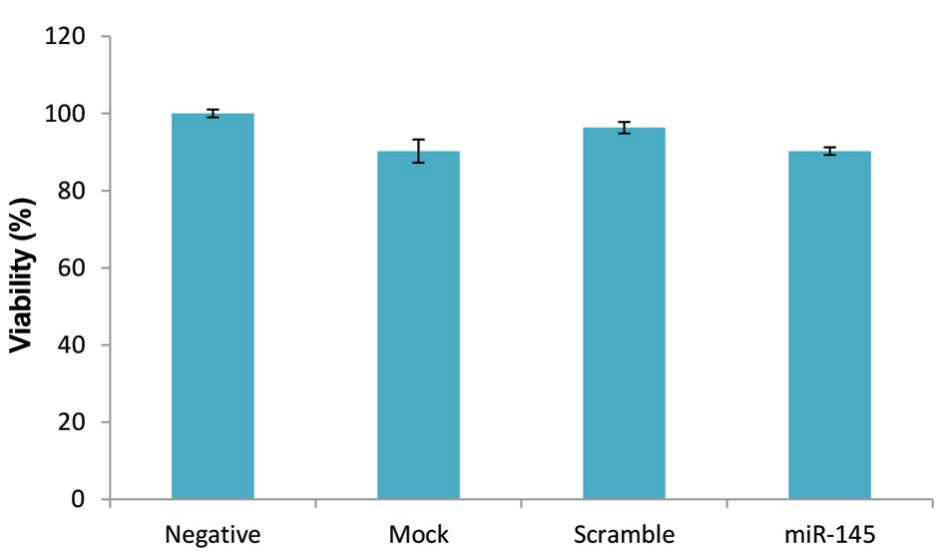
Viability of peripheral blood mononuclear cells (PBMCs) after transfection of mimic miR-145-5p was analyzed by MTT assay. The optimal density of each well was determined at 590 nm and viability didn’t have significant difference between groups.

MTT assay was performed in order to assess the cytotoxicity of the transfection process. Our results showed that miR-145-5p mimic has no significant cytotoxic effect on PBMCs (P=0.416) and a viability of 90% was observed in the test group (Fig .6). 

## Discussion

Regulation of the immune system and homeostasis is vital for prevention of pathological processes that lead to autoimmune diseases (26). In this context, modulatory molecules like SEMA3A and miRNAs have a pivotal role in induction and maintenance of self-tolerance. As mentioned earlier, SEMA3A acts as a terminator of T cells and B cell activity and down-regulates the expression of pro-inflammatory cytokines (23). So, decreased *SEMA3A* expression may lead to inflammatory conditions. 

Some studies have shown that *SEMA3A* is down- regulated in some autoimmune diseases such as SLE, RA, SSc and psoriasis. As previously reported, administration of SEMA3A could decrease anti-collagen IgG and inhibit proinflammatory cytokines such as IL-17 and IFN-. in RA. On the other hand, it can elevate IL-10 concentration in the serum (23). Moreover, it has been suggested that SEMA3A could be regarded as a diagnostic marker for SLE disease activity and renal damage (7). In accordance with these findings, in our previous study, we showed that *SEMA3A* expression was reduced in PBMCs of relapsing-remitting multiple sclerosis (RRMS) patients (27). In addition, in a study done by Gutiérrez -Franco and et al on the EAE mice, a decrease in *SEMA3A* expression level in the immune system was revealed. Their results indicated that the levels of the protein expression were down-regulated following EAE induction but *SEMA3A* gene expression did not differ significantly during EAE development. They reported that the expression of *SEMA3A* might be controlled at a post-transcriptional level (28). In order to consider SEMA3A as a therapeutic target, the reasons underlying its down-regulation must become clear. Therefore, we aimed to investigate the effect of miR-145 as a post-transcriptional epigenetic factor. 

By applying bioinformatic tools, we observed that miR-145-5p might be able to silence SEMA3A with a probability of 99%. Also, information obtained from these databases indicted that the silencing effect of miR-145-5p on *SEMA3A* had not been experimentally confirmed, so far. Moreover, it was previously demonstrated that miR145- 5p is up-regulated in multiple sclerosis and primary biliary cirrhosis (22, 29). Therefore, based on these data and the bioinformatic predictions, we investigated the relation between *SEMA3A* expression and miR-145-5p inhibitory effect.

The current study showed that *SEMA3A* is negatively regulated by miR-145-5p. Based on qRT-PCR analysis, miR-145-5p degenerated *SEMA3A* mRNA and led to down-regulation of *SEMA3A* expression. We also assessed SEMA3A concentration in culture supernatant before and after transfection of miR-145-5p mimic. SEMA3A secretion level by PBMCs was significantly reduced in the transfected group compared to control groups. So, these findings confirmed our previous bioinformatic prediction of inhibitory effect of miR-145-5p on *SEMA3A* expression. 

Based on our literature review, there was no study on miR-145-5p effect on *SEMA3A* in autoimmune diseases to make a comparison with our results. However, alteration of miR-145 has been investigated in some autoimmune diseases and cancers.

As mentioned before, miR-145 expression was up- regulated in MS and primary biliary cirrhosis (22, 29). However, in another study, it was stated that miR-1455p is down-regulated in SLE patients (30). Controversial results reported by these studies may be explained by the differences in immunopathogenesis of these diseases as Th2 cells are more highlighted in SLE.

The significant increase in miR-145 and decrease in SEMA3A in PBMCs of MS patients might be explained by our results. In this study, we did not investigate the exact mechanism underlying this down-regulation, but we concluded that administration of miR-145 leads to decreased expression of *SEMA3A*. So, inadequate SEMA3A production results in excessive activity of immune cells and exacerbation of inflammation. 

It is suggested that more investigations should be done to clear the correlation between immune regulators like SEMA3A and other miRNAs which are altered in T or B cells and these findings can help to open up a new insight about immune regulation.

## Conclusion

Our results revealed that miR-145-5pcan down- regulate *SEMA3A* expression. Decreased expression of *SEMA3A*, which is observed in some autoimmune disorders, may lead to over-activation of T cells, B cells and increased production of pro-inflammatory cytokines. This reduced expression might be due to over-expression of miRNAs like miR-145-5p. Thus, our data may shed light on a less determined aspect of immune system homeostasis and may recommend for a probable therapeutic approach for some autoimmune disorders in the future.

## References

[1] Eixarch H, Gutiérrez-Franco A, Montalban X, Espejo C (2013). Semaphorins 3A and 7A: potential immune and neuroregenerative targets in multiple sclerosis. Trends Mol Med.

[2] Yazdani U, Terman JR (2006). The semaphorins. Genome Biol.

[3] Luo Y, Raible D, Raper JA (1993). Collapsin: a protein in brain that induces the collapse and paralysis of neuronal growth cones. Cell.

[4] Vadasz Z, Toubi E (2014). Semaphorins: their dual role in regulating immune-mediated diseases. Clin Rev Allergy Immunol.

[5] Lepelletier Y, Moura IC, Had-Slimane R, Renand A, Fiorentino S, Baude C (2006). Immunosuppressive role of semaphorin-3A on T cell proliferation is mediated by inhibition of actin cytoskeleton reorganization. Eur J Immunol.

[6] Vadasz Z, Haj T, Kessel A, Toubi E (2013). B-regulatory cells in autoimmunity and immune mediated inflammation. FEBS Lett.

[7] Vadasz Z, Haj T, Halasz K, Rosner I, Slobodin G, Attias D (2012). Semaphorin 3A is a marker for disease activity and a potential immunoregulator in systemic lupus erythematosus. Arthritis Res Ther.

[8] Okuno T, Nakatsuji Y, Kumanogoh A (2011). The role of immune semaphorins in multiple sclerosis. FEBS Lett.

[9] Bruder D, Probst-Kepper M, Westendorf AM, Geffers R, Beissert S, Loser K (2004). Neuropilin-1: a surface marker of regulatory T cells. Eur J Immunol.

[10] Moretti S, Procopio A, Lazzarini R, Rippo MR, Testa R, Marra M (2008). Semaphorin3A signaling controls Fas (CD95)-mediated apoptosis by promoting Fas translocation into lipid rafts. Blood.

[11] Pauley KM, Cha S, Chan EK (2009). MicroRNA in autoimmunity and autoimmune diseases. J Autoimmun.

[12] Peschansky VJ, Wahlestedt C (2014). Non-coding RNAs as direct and indirect modulators of epigenetic regulation. Epigenetics.

[13] Bartel DP (2004). MicroRNAs: genomics, biogenesis, mechanism, and function. Cell.

[14] Saki N, Abroun S, Soleimani M, Hajizamani S, Shahjahani M, Kast RE (2015). Involvement of MicroRNA in T-Cell Differentiation and Malignancy. Int J Hematol Oncol Stem Cell Res.

[15] Iorio MV, Croce CM (2009). MicroRNAs in cancer: small molecules with a huge impact. J Clin Oncol.

[16] Medrano S, Sequeira-Lopez ML, Gomez RA (2014). Deletion of the miR143/145 cluster leads to hydronephrosis in mice. Am J Pathol.

[17] Sachdeva M, Mo YY (2012). MIR145 (microRNA 145). Atlas Genet Cytogenet Oncol Haematol.

[18] Sachdeva M, Zhu S, Wu F, Wu H, Walia V, Kumar S (2009). p53 represses c-Myc through induction of the tumor suppressor miR-145. Proc Natl Acad Sci India Sect B Biol Sci.

[19] Sachdeva M, Mo YY (2010). MicroRNA-145 suppresses cell invasion and metastasis by directly targeting mucin 1. Cancer Res.

[20] Fayyad-Kazan H, Rouas R, Fayyad-Kazan M, Badran R, El Zein N, Lewalle P (2012). MicroRNA profile of circulating CD4-positive regulatory T cells in human adults and impact of differentially expressed microRNAs on expression of two genes essential to their function. J Biol Chem.

[21] Guerau-de-Arellano M, Alder H, Ozer HG, Lovett-Racke A, Racke MK (2012). miRNA profiling for biomarker discovery in multiple sclerosis: from microarray to deep sequencing. J Neuroimmunol.

[22] Ma X, Zhou J, Zhong Y, Jiang L, Mu P, Li Y (2014). Expression, regulation and function of microRNAs in multiple sclerosis. Int J Med Sci.

[23] Catalano A (2010). The neuroimmune semaphorin-3A reduces inflammation and progression of experimental autoimmune arthritis. J Immunol.

[24] Kou K, Nakamura F, Aihara M, Chen H, Seto K, Komuri-Yamaguchi J (2012). Decreased expression of semaphorin-3A, a neurite-collapsing factor, is associated with itch in psoriatic skin. Acta Derm Venereol.

[25] Rimar D, Nov Y, Rosner I, Slobodin G, Rozenbaum M, Halasz K (2015). Semaphorin 3A: an immunoregulator in systemic sclerosis. Rheumatol Int.

[26] Dai R, Ahmed SA (2011). MicroRNA, a new paradigm for understanding immunoregulation, inflammation, and autoimmune diseases. Transl Res.

[27] Rezaeepoor M, Shapoori S, Ganjalikhani-hakemi M, Etemadifar M, Alsahebfosoul F, Eskandari N (2017). Decreased expression of Sema3A, an immune modulator, in blood sample of multiple sclerosis patients. Gene.

[28] Gutiérrez-Franco A, Costa C, Eixarch H, Castillo M, Medina-Rodríguez EM, Bribián A (2016). Differential expression of sema3A and sema7A in a murine model of multiple sclerosis: Implications for a therapeutic design. Clin Immunol.

[29] Padgett KA, Lan RY, Leung PC, Lleo A, Dawson K, Pfeiff J (2009). Primary biliary cirrhosis is associated with altered hepatic microRNA expression. J Autoimmun.

[30] Lu MC, Lai NS, Chen HC, Yu HC, Huang KY, Tung CH (2013). Decreased microRNA (miR)-145 and increased miR-224 expression in T cells from patients with systemic lupus erythematosus involved in lupus immunopathogenesis. Clin Exp Immunol.

